# Organisation und Umsetzung des Arbeitsschutzes in Kitas

**DOI:** 10.1007/s40664-021-00454-6

**Published:** 2022-02-04

**Authors:** Martina Michaelis, Ulrich Stößel, Frank Bieler, Heike Schambortski, Albert Nienhaus

**Affiliations:** 1 FFAS – Freiburger Forschungsstelle Arbeits- und Sozialmedizin, Bertoldstr. 63, 79098 Freiburg, Deutschland; 2 Gesamtbereich Präventionsdienste Hauptverwaltung, Berufsgenossenschaft für Gesundheitsdienst und Wohlfahrtspflege (BGW), Hamburg, Deutschland; 3 Abteilung Arbeitsmedizin, Gefahrstoffe, Gesundheitswissenschaften (AGG), Berufsgenossenschaft für Gesundheitsdienst und Wohlfahrtspflege (BGW), Hamburg, Deutschland

**Keywords:** Arbeitsschutzregelungen, Umsetzungsgrad, Kindertagesstätten, Standardisierte Erhebung, Befragung von Leitungspersonen, Occupational health and safety regulations, Degree of implementation, Day care centers, Standardized survey, Survey of management personnel

## Abstract

**Hintergrund und Fragestellung:**

Physische und psychische Belastungen sowie Infektionsgefährdungen von Beschäftigten in Kindertagesstätten (Kitas) machen die Einhaltung staatlicher und berufsgenossenschaftlicher Arbeitsschutzregelungen essenziell. Wie gut der Arbeitsschutz hier organisiert ist und Maßnahmen auch tatsächlich umgesetzt werden, ist empirisch bislang nicht bekannt. Diese Lücke soll mit einer Bestandsaufnahme geschlossen werden.

**Material und Methoden:**

In der zweiten Jahreshälfte 2020 wurden 120 zufällig ausgewählte Kita-Leitungen in ganz Deutschland meist kirchlicher Mitgliedsbetriebe der Berufsgenossenschaft für Gesundheitsdienst und Wohlfahrtspflege (BGW) interviewt. Dies geschah durch Angehörige der BGW-Präventionsdienste mittels eines standardisierten Erhebungsinstruments. Die erhobenen Aspekte (mehrheitlich zur Organisation) wurden in einem standardisierten Summenindex zwischen0 und 1 (schlechtester bis bester Arbeitsschutz) zusammengefasst und deskriptiv ausgewertet.

**Ergebnisse:**

Die Anforderungen an die *Organisation* des Arbeitsschutzes sind vielfach erfüllt. Für eine tatsächliche *Umsetzung *organisierter Arbeitsschutzvorgaben sind noch gewisse Entwicklungsmöglichkeiten erkennbar. Der standardisierte Summenindex beträgt 0,82 (Standardabweichung: 0,16). Zwei Drittel der Befragten schätzten den Arbeitsschutz in ihrer Kita als insgesamt gut/sehr gut ein. In Freitexten wurde häufig der Wunsch nach mehr Informationen zum Arbeitsschutz und besserer entsprechender Kommunikation des Trägers geäußert.

**Diskussion und Ausblick:**

Die Ergebnisse bestätigen eine höhere *formale* Qualität des Arbeitsschutzes als in anderen – branchenübergreifenden – Studien in kleineren Betrieben. Zur kontinuierlichen Verbesserung der tatsächlichen *Umsetzung* organisierter Maßnahmen können – in Kitas bislang noch selten genutzte – Arbeitsschutzmanagementsysteme unterstützend wirken. Es stehen entsprechende Instrumente zur Verfügung, für deren Einsatz in Zukunft mehr geworben werden sollte. Limitationen der Studie sind u. a. mögliche Verzerrungen durch Antworten an Vertreter einer Institution, die auch für die Überwachung des Arbeitsschutzes zuständig ist.

**Zusatzmaterial online:**

In der Online-Version dieses Artikels (10.1007/s40664-021-00454-6) finden Sie Tabelle S1: Konstruktion eines standardisierten Summenindex.

## Abstract (en)

**Background and research question:**

Physical and mental stress as well as infection hazards of employees in child day care centers require compliance with governmental occupational health and safety (OHS) regulations. How well OHS is organized and how measures are actually implemented are not yet empirically known. This gap was closed with an epidemiological study on the status quo.

**Material and methods:**

In the second half of 2020 a total of 120 managers of day care centers in Germany, mostly church-run, which are member companies of the German Statutory Accident Insurance and Prevention in the Health and Welfare Services (BGW), were randomly selected and interviewed. This was done by members of the BGW prevention services using a standardized survey instrument. The surveyed aspects (mostly concerning the OHS organization) were summarized in a standardized sum index between 0 and 1 (worst to best occupational health and safety) and analyzed descriptively.

**Results:**

The requirements for the organization of OHS were fulfilled in many cases. There is still some potential left for improvement in the actual implementation of organized OHS requirements. The standardized sum index is 0.82 (standard deviation 0.16). Of the respondents two thirds rated OHS in their day care center as good/very good in a global item. In free texts, the desire for more information on OHS and better communication between the day care center and the respective provider was frequently expressed.

**Discussion and outlook:**

The results confirmed a higher formal quality of OHS as found in other cross-sectional studies. To continuously improve the actual implementation of organized measures, OHS management systems, which are still rarely used in day care centers, could have a supporting effect. Corresponding instruments are available and their use should be more strongly promoted in the future. Limitations of the study include possible bias due to responses to representatives of an institution that is also responsible for OHS inspections.

In standardisierten aufsuchenden Befragungen von Leitungspersonen in Kindertagesstätten mit meist kirchlichen Trägern wurde durch Vertreter der berufsgenossenschaftlichen Präventionsdienste ermittelt, inwieweit staatliche Arbeitsschutzregeln organisiert und umgesetzt werden.

Die Arbeit in Kindertagesstätten (Kitas) ist gekennzeichnet durch körperliche und psychische Belastungen sowie Infektionsgefährdungen [11, 16, 17]. Trotz des Ausbaus von Einrichtungen in den letzten Jahren und des damit verbundenen Personalzuwachses [2] besteht nach wie vor ein Mangel an Fachkräften, verbunden mit psychosozialen Folgen, wie u. a. im Rahmen der Arbeitsorganisation und der sozialen Beziehungen [2, 10]. Die Organisation und Einhaltung von Arbeitsschutzvorgaben sind deshalb essenziell.

Wie staatlich und berufsgenossenschaftlich vorgeschriebener Arbeitsschutz in Kitas organisiert ist und inwiefern die Maßnahmen auch tatsächlich umgesetzt werden, ist empirisch bislang nicht untersucht. Diese Lücke sollte mit der hier vorgestellten explorativen Bestandsaufnahme geschlossen werden. Ziel war die Identifikation von Aspekten, für welche die Einrichtungen bzw. Träger möglicherweise mehr unterstützende Beratung durch die Präventionsdienste der zuständigen Berufsgenossenschaft für Gesundheitsdienst und Wohlfahrtspflege (BGW) benötigen.

## Methoden

### Zugang zum Kollektiv

Es wurden deutschlandweit 120 Mitgliedsbetriebe der Berufsgenossenschaft für Gesundheitsdienst und Wohlfahrtspflege (BGW) zufällig ausgewählt und zwischen Juli 2020 und Januar 2021 von Angehörigen der BGW-Präventionsdienste aus 7 Bezirksstellen aufgesucht (Berlin, Bochum, Delmenhorst, Hamburg, Hannover, Karlsruhe, Köln, Mainz, München, Würzburg). Dabei erfolgten standardisierte Interviews mit Kita-Leitungen zur Situation des Arbeitsschutzes. Diese wurden zuvor über den Zweck der Studie und die anonymisierte Auswertung einer Nicht-BGW-Organisation informiert. Die Interviews waren freiwillig und eingebettet in einen üblichen Beratungsbesuch der Präventionsdienste.

### Erhebungsinstrument

Grundlage der Interviews war ein standardisierter Fragebogen zur Organisation und Umsetzung des Arbeitsschutzes. Hinsichtlich der *Arbeitsschutzorganisation *wurden Teile der Basisversionen der *ORGAchecks* der Gemeinsamen Deutschen Arbeitsschutzstrategie (GDA; Bausteine 1–6) und der BGW (Bausteine 9, 11, 15) verwendet [3, 6]. Sie wurden durch selbstentwickelte Fragen zur *Umsetzung* der Vorgaben ergänzt. Inhalte waren neben Arbeitsschutzstrukturen auch die Organisation und Umsetzung von 8 weiteren Aspekten (Gefährdungsbeurteilungen, arbeitsmedizinische Vorsorge, Benennung von Sicherheitsbeauftragten, Erste-Hilfe- bzw. Notfallmaßnahmen, Unterweisungen bzw. Arbeitsschutzqualifizierung von Beschäftigen, Arbeitsschutzkontrolle) untersucht. Ergänzend dazu wurde eine Selbsteinschätzung der Arbeitsschutzqualität in der eigenen Kita als Schulnote von 1 (sehr gut) bis 6 (ungenügend) sowie Wünsche und Verbesserungen zum Arbeitsschutz erfragt.

### Stichprobenauswahl und Datenschutz

Die zufällige Auswahl von 120 Kitas mit mehrheitlich kirchlichen Trägern in ganz Deutschland (Grundgesamtheit 11.000 BGW-Mitgliedsbetriebe) erfolgte durch die BGW, stratifiziert nach katholischen und evangelischen/freien/öffentlichen Trägern im Verhältnis 1:1. Die ausgefüllten Bögen wurden der datenauswertenden Stelle (Freiburger Forschungsstelle Arbeits- und Sozialmedizin [FFAS]) pseudonymisiert zugeleitet.

### Datenauswertung und statistische Methoden

Die wesentlichen Variablen aus 9 Organisationsbereichen sowie weitere 6 Variablen im Zusammenhang mit der Umsetzung des Arbeitsschutzes wurden in einem standardisierten Summenindex zwischen 0 und 1 (schlechtester bis bester Arbeitsschutz) zusammengefasst. Einzelheiten sind der Tabelle S1 des Zusatzmaterials online zu entnehmen. Die Datenauswertung erfolgte deskriptiv mittels Prozent- bzw. Mittelwerten und Standardabweichungen (SD). Der Zusammenhang zwischen dem Summenindex und der Selbsteinschätzung der Arbeitsschutzqualität erfolgte mittels Rangkorrelationskoeffizienten Spearmans Rho und den dazugehörigen Effektstärke-Cut-off-Werten 0,15, 0,25 und 0,50 für einen kleinen, moderaten oder starken Zusammenhang [7]. Fehlende Angaben wurden von den Analysen ausgeschlossen.

## Ergebnisse

### Datenrücklauf/-qualität und Stichprobenmerkmale

Es wurden die Daten von insgesamt 114 Kitas mit einem kirchlichen und 6 mit einem freien oder öffentlichen Träger ausgewertet (insgesamt *n* = 120). Die Ausfüllqualität der Erhebungsbögen war gut, fehlende Angaben lagen in der Regel bei höchstens 5 %. Ausnahmen waren die Frage, ob die Einsatzzeiten von bestellten Fachkräften für Arbeitssicherheit und Betriebsärzten in Kitas > 10 Beschäftigte ermittelt wurden, und die Frage nach der Häufigkeit von Arbeitsschutzausschusstreffen (Tab. 1) sowie nach der Umsetzung und Wirksamkeitsprüfung von Maßnahmen, die als Ergebnis von Gefährdungsbeurteilungen festgelegt wurden (Tab. 2).

In 93,3 % (*n* = 112) der Kitas wurde die Kita-Leitung interviewt, in den übrigen die stellvertretende Leitung (*n* = 6), eine pädagogische Fachkraft oder ein Trägervertreter. Hinsichtlich ihrer Größe wurden im Mittel 75 Kinder (SD 35) mit im Mittel 4 Gruppen (SD 2) betreut. In der Kita waren durchschnittlich 13,7 (SD 7,6) pädagogische Fachkräfte beschäftigt. Weitere Merkmale der Kitas zeigt Tab. 1.

**Table 1:** 

**–**	–	Prozent	*N* (ja)	*N* (gültig)
Art der Kinderbetreuung (Mehrfachantworten)	Krippe	70,8	85	120
	Kindergarten	97,5	117	–
	Hort	8,3	10	–
	Integrations-Kita	36,7	44	–
Betriebliche Interessenvertretung vorhanden	Ja	69,2	83	120
Wenn ja (*n* = 83): Beteiligung bei Arbeitsschutzthemen	Ja	80,0	28	35 von 83
*Anzahl Beschäftigte für Bestellung eines ...*	–	–	–	–
Arbeitsschutzausschusses (ASA)^a^	*n* > 20 Beschäftigte	14,5	17	117
Sicherheitsbeauftragten^b^	> 20 Beschäftigte + Kinder	99,2	117	118
Kita-Arbeitsschutzbetreuungsmodell nach DGUV-Vorschrift 2	Regelbetreuung ≤ 10 Beschäftigte	32,5	39	120
	Regelbetreuung > 10 Beschäftigte	65,0	78	–
	Alternative Betreuung („Unternehmermodell“)	2,5	3	–

^a^ gemäß §11 Arbeitssicherheitsgesetz (ASiG)

^b^ gemäß §22(1) und §2(1)Nr. 8a, Sozialgesetzbuch (SGB) 7

### Arbeitsschutzstrukturen

In 8,5 % (*n* = 10) der Kitas wird ein Arbeitsschutzmanagementsystem [5, 9] genutzt. Bei diesem werden die Themen des Arbeits- und Gesundheitsschutzes planmäßig, zielorientiert und systematisch in die betriebliche Gesamtorganisation integriert. Sieben dieser 10 Befragten meinten, dass sich dadurch der Arbeitsschutz auch verbessert habe. 10,0 % (*n* = 11 von 110 verbleibenden) wussten, dass ein solches System in Zukunft genutzt werden soll, 17,3 % (*n* = 19), dass es nicht genutzt werden soll und 38,2 % (*n* = 80) konnten die Perspektive nicht einschätzen.

Die betriebsärztliche und sicherheitstechnische Betreuung erfolgt bei zwei Dritteln der Kitas als Regelbetreuung für Betriebe über 10 Beschäftigte. In der überwiegenden Mehrheit (über 85 %) der Kitagruppen mit einem Regelarbeitsschutzbetreuungsmodell wurden sowohl Fachkräfte für Arbeitssicherheit (FaSis) als auch Betriebsärzte schriftlich beauftragt (≤ 10 Beschäftigte) oder bestellt (> 10 Beschäftigte; Tab. 2).

**Table 2:** 

**–**	**–**	Regelbetreuung für:^a^			
**–**	**–**	b) ≤ 10 Beschäftigte (*n* = 39)		b) > 10 Beschäftigte (*n* = 78)	
Aspekt (Datenbasis)	**–**	*N* (%)	*N*_(gültig)_	*N* (%)	*N*_(gültig)_
FaSi/Betriebsarzt schriftlich a) beauftragt bzw. b) bestellt	Ja	39 (100,0 %)	39	72 (92,3 %)	78
Wenn a) beauftragt (*n* = 38) bzw. b) bestellt (*n* = 72): vollständig?	FaSi und Betriebsarzt	33 (86,8 %)	38	66 (94,3 %)	70
	Betriebsarzt (nur)	5 (13,2 %)	–	2 (2,9 %)	–
	FaSi (nur)	0 (0,0 %)	–	2 (2,9 %)	–
Wenn beauftragt (*n* = 38): beteiligt bei besonderen Anlässen^b^	Ja	23 (67,6 %)	34	–	–
Wenn beauftragt: an der Erstellung/Durchführung von Gefährdungsbeurteilungen beteiligt	FaSi [*n* = a) 33 bzw. b) 68]	21 (30,9 %)	68	13 (19,1 %)	68
	Betriebsarzt [*n* = a) 68 bzw. b) 68]	10 (26,3 %)	38	21 (17,6 %)	68
Wenn bestellt (*n* = 72):	Einsatzzeiten für die Grundbetreuung ermittelt	–	–	35 (60,3 %)	58
–	Zusätzliche Aufgaben für die betriebsspezifische Betreuung ermittelt und umgesetzt	–	–	31 (53,4 %)	58
Wenn a) beauftragt bzw. b) bestellt: schriftlicher Bericht der Arbeitsschutzakteure über Tätigkeiten und Ergebnisse ^c^	FaSi [*n* = a) 33 bzw. b) 68]	14 (42,4 %)	33	32 (47,1 %)	68
	Betriebsarzt [*n* = a) 33 bzw. b) 68]	16 (42,1 %)	38	36 (52,9 %)	68
Arbeitsschutzausschuss (ASA) vorhanden (*n* = 78)^d^	Ja	–	–	31 (40,3 %)	77
Wenn ja (*n* = 31): Häufigkeit der ASA-Treffen^d^	Mindestens vierteljährlich	–	–	12 (54,5 %)	22
	Regelmäßig, aber seltener	–	–	10 (45,5 %)	–

*FaSi* Fachkraft für Arbeitssicherheit

^a^ Betriebsärztliche und sicherheitstechnische Regelbetreuung; schriftliche a) Beauftragung ≤ 10 Beschäftigte bzw. b) Bestellung > 10 Beschäftigte; keine weitere Auswertung für *n* = 3 Kitas mit sog. alternativer Betreuung

^b^ Zum Beispiel Einführung neuer Arbeitsmittel, neuer Arbeitsverfahren, berufsbedingte Erkrankungen, Arbeitsunfälle

^c^ Sofern beauftragt/bestellt

^d^ Bezogen auf alle Kitas, unabhängig davon, ob ein ASA überhaupt erforderlich ist (dies gilt für *n* = 17 Kitas > 20 Beschäftigte), da viele Befragte von einem ASA berichteten. Bei 10 von 17 Kitas mit > 20 Beschäftigten existierte kein ASA

Weitere Einzelheiten für Kitas mit einer betriebsärztlichen und sicherheitstechnischen Regelbetreuung (unter und über 10 Beschäftigte) lassen sich gemäß Tab. 2 wie folgt zusammenfassen:

- *Kitas mit einer Regelbetreuung ≤* *10 Beschäftigte*: In zwei Dritteln der Einrichtungen werden die genannten Arbeitsschutzakteure auch bei besonderen Anlässen, z. B. zur Einführung neuer Arbeitsverfahren, beteiligt. Gleiches gilt für FaSis bei der Erstellung und Aktualisierung von Gefährdungsbeurteilungen, deutlich weniger für Betriebsärzte.
- *Kitas mit einer Regelbetreuung >* *10 Beschäftigte: *Die Einsatzzeiten für die Grundbetreuung sowie für zusätzliche Aufgaben im Rahmen der betriebsspezifischen Betreuung durch FaSis und Betriebsarzt werden in rund 50 % der Kitas ermittelt (Anteil fehlender Angaben hier allerdings 19 %). Die Existenz eines Arbeitsschutzausschusses (ASA) nannten 59 % aller Befragten. Die Antworten waren unabhängig davon, ob ein ASA gemäß §11 ASiG überhaupt erforderlich ist (bei > 20 Beschäftigten). Von *n* = 17 dieser vergleichsweise größeren Kitas mit entsprechender ASA-Erfordernis hatten nur 10 einen ASA. Aus den Kitas, die von einem ASA berichteten, erwähnte rund die Hälfte ein mindestens vierteljährliches, die andere Hälfte seltenere Treffen des Arbeitskreises (Anteil fehlender Angaben liegt hier allerdings bei 29 %).

Ebenfalls die Hälfte der Arbeitsschutzakteure berichtet schriftlich über ihre Aktivitäten.

### Sicherheitsbeauftragter

Für Kitas mit mindestens 20 Personen (wobei Kinder gemäß §11 Arbeitssicherheitsgesetz in diesem Fall mitgezählt werden; *n* = 117) ist die schriftliche Benennung eines Sicherheitsbeauftragten erforderlich. Es wurde in 87,2 % (*n* = 102) der Kitas eine fachlich als geeignet befundene Person benannt, wenn auch nur in 67,3 % Einrichtungen (*n* = 68) schriftlich.

Der/die Beauftragte ist in 69,4 % „immer“ und in 20,4 % „meistens“ an Arbeitsschutzthemen beteiligt (98 Kitas mit gültigen Antworten von 102).

### Gefährdungsbeurteilungen

In insgesamt 93,3 % der Kitas werden Gefährdungsbeurteilungen organisiert (Tab. 3), in 75,6 % „vollständig“ oder „überwiegend“. Rund 50 % aller Kitas, in denen Gefährdungsbeurteilungen organisiert sind, werden diese auch regelmäßig für alle Bereiche durchgeführt.

Fast immer werden die Vorgänge schriftlich dokumentiert und im Bedarfsfall auch Maßnahmen zur Gefährdungsreduktion ergriffen; diese werden in rund 75 % auch „immer“ oder „meistens“ umgesetzt.

**Table 3:** 

Aspekt (Datenbasis)	Ausprägung	*N* (%)	*N*_(gültig)_
Gefährdungsbeurteilungen organisiert^a^	Vollständig	51 (42,9 %)	119
	Überwiegend	39 (32,8 %)	–
	Überwiegend nicht	21 (17,6 %)	–
	Gar nicht	8 (6,7 %)	–
Wenn organisiert (*n* = 111): ^b^	Regelmäßig für alle Bereiche	48 (56,3 %)	10.303
	Regelmäßig, aber nicht für alle Bereiche	17 (16,5 %)	–
	Nur unregelmäßig, aber für alle Bereiche	9 (8,7 %)	–
	Unregelmäßig und auch für nicht alle Bereiche	18 (17,5 %)	–
	Nur aus besonderen Gründen	1 (1,0 %)	–
Wenn organisiert: (*n* = 111):	Gefährdungsbeurteilung dokumentiert	91 (89,2 %)	103
	Maßnahmen festgelegt	91 (90,1 %)	101
Wenn Maßnahmen festgelegt (*n* = 91): Umsetzung erfolgt	Immer	34 (42,0 %)	81
	Meistens	39 (48,1 %)	–
	Manchmal	8 (9,9 %)	–
	Nie	0 (0,0 %)	–
Wenn Maßnahmen festgelegt (*n* = 91): Wirksamkeit überprüft	Immer	33 (41,3 %)	80
	Meistens	27 (33,8 %)	–
	Manchmal	18 (22,5 %)	–
	Nie	2 (2,5 %)	–

^a^ Benennung von Zuständigkeiten, Beteiligungen, Art und Zeitpunkt, Aktualisierung/Überprüfung)

^b^ Vollständig, überwiegend bzw. überwiegend nicht organisiert (*n* = 111)

### Arbeitsmedizinische Vorsorge

Nahezu immer (95,8 %) ist die arbeitsmedizinische Vorsorge organisiert, d. h. Zuständigkeiten, Definition des Personenkreises, des Anlasses, der Intervalle, der Information von Beschäftigten sowie der Durchführung und Kontrolle werden benannt; in 83,3 % als „vollständig“ oder „überwiegend“.

Sofern sie organisiert ist, erfolgt eine *Pflichtvorsorge* in 76,6 % von 111 Kitas „regelmäßig“, in 13,5 % „unregelmäßig“ und in 9,9 % „nie“. In 87,9 % der Kitas, in der sie organisiert ist (*n* = 100), werden nur solche Beschäftigte mit risikoreichen Tätigkeiten betraut, die den Vorsorgetermin beim betriebsärztlichen Dienst wahrgenommen haben.

Über die Möglichkeit einer *Angebotsvorsorge* werden 74,1 % der Beschäftigten in Kitas, in der die arbeitsmedizinische Vorsorge organisiert ist, auch informiert, 46,4 % regelmäßig. Gleiches gilt für die entsprechende Durchführung.

### Erste Hilfe und Notfallmaßnahmen

Die erforderlichen Erste-Hilfe-Maßnahmen sind zu 91 % „vollständig“ organisiert, Notfallmaßnahmen zu 75 % (Tab. 4). In nahezu allen Kitas wurden auch Erst- und in 68 % Brandschutzhelfer ausgebildet. Analog hierzu sind die entsprechenden Maßnahmen „allen“ oder „den meisten“ Beschäftigten bekannt.

**Table 4:** 

–	–		Erste Hilfe		Notfallmaßnahmen		
Aspekt	Ausprägung	*N* (%)		*N*_(gültig)_		*N* (%)	*N*_(gültig)_
Maßnahmen organisiert^a,b^	Vollständig	109 (90,8 %)		120		90 (75,0 %)	120
	Überwiegend	11 (9,2 %)		–		25 (20,8 %)	–
	Überwiegend nicht	0 (0,0 %)		–		3 (2,5 %)	–
	Gar nicht	0 (0,0 %)		–		2 (1,7 %)	–
Erst- bzw. Brandschutzhelfer aus- und weitergebildet	Ja	118 (98,3 %)		120		80 (67,8 %)	118
Vorgehensweisen der Ersten Hilfe bzw. bei Notfällen bekannt^c^	Allen Beschäftigten	110 (91,7 %)		120		91 (75,8 %)	120
	Den meisten	9 (7,5 %)		–		22 (18,3 %)	–
	Nur wenigen	1 (0,8 %)		–		5 (4,2 %)	–
	Niemand	0 (0,0 %)		–		2 (1,7 %)	–

^a^ Erste Hilfe: Zuständigkeiten, Planung/Abläufe, Anzahl, Benennung von Ersthelfer, Rettungskette, Hilfsmittel, Aufzeichnungen über Erste Hilfe, Information

^b^ Notfallmaßnahmen, z. B. Brände: Zuständigkeiten, Planung/Abläufe, Rettungskette, Hilfsmittel

^c^ Zum Beispiel Rettungskette bekannt geben, regelmäßige Brandschutz- bzw. Rettungsübungen

### Arbeitsschutzunterweisungen, Arbeitsschutzqualifikation und -kontrollen

Fast immer wird die Unterweisung von Beschäftigten nach §12 Arbeitsschutzgesetz organisiert (96,7 %), mehrheitlich auch „vollständig“ oder „überwiegend“. Das Gleiche gilt, wenn es darum geht, den Qualifizierungsbedarf für alle mit Arbeitsschutzaufgaben Betrauten zu ermitteln; auch dies erfolgte in der Hälfte der Kitas „regelmäßig“. Analog hierzu werden entsprechende Qualifizierungsmaßnahmen auch durchgeführt.

Die meisten Kita-Leitungen gaben an, dass ihnen die vom Arbeitgeber übertragenen Arbeitsschutzpflichten nach §13 ArbSchG (zumindest im Groben) auch bekannt sind.

Ebenfalls wird in fast allen Kitas auch das Einhalten des Arbeitsschutzes kontrolliert, in 81 % „vollständig“ oder „überwiegend“. Dies erfolgt entweder durch den Arbeitgeber, durch den/die Arbeitsschutzkoordinator oder die Kita-Leitung selbst. Nach Kontrollen erfolgen bei Bedarf in den meisten Kitas auch Verbesserungen; in der Hälfte der Kitas „immer“ (Tab. 5).

**Table 5:** 

Aspekt (Datenbasis)	Ausprägung	*N* (%)	*N*_(gültig)_
Unterweisung für alle organisiert ^a^	Vollständig	67 (58,8 %)	114
	Überwiegend	29 (25,4 %)	–
	Überwiegend nicht	14 (12,3 %	–
	Gar nicht	4 (3,5 %)	–
Qualifizierungsbedarf für alle mit Arbeitsschutzaufgaben Betrauten ermittelt	Regelmäßig	59 (49,6 %)	119
	Unregelmäßig	49 (41,2 %)	–
	Nie	11 (9,2 %)	–
Qualifizierungsmaßnahmen durchgeführt	Regelmäßig	65 (55,6 %)	117
	Unregelmäßig	41 (35,0 %)	–
	Nie	11 (9,4 %)	–
Kita-Leitung sind Arbeitsschutzpflichten nach §13 Arbeitsschutzgesetz bekannt	Ja	95 (79,8 %)	119
Arbeitsschutz regelmäßig durch Arbeitgeber und Kita-Leitung kontrolliert	Vollständig	51 (42,5 %)	120
	Überwiegend	46 (38,3 %)	–
	Überwiegend nicht	19 (15,8 %)	–
	Gar nicht	4 (3,3 %)	–
Wenn kontrolliert (*n* = 116): Verbesserungen bei Bedarf umgesetzt	Immer	58 (50,9 %)	114
	Meistens	42 (36,8 %)	–
	Manchmal	13 (11,4 %)	–
	Nie	1 (0,9 %)	–

^a^ Themen, Zuständigkeit, Beteiligung, Methoden, Anlass/Intervall (mind. einmal jährlich), Aktualisierung, Überprüfung

### Arbeitsschutz-Summenindex und Selbsteinschätzung der Arbeitsschutzqualität in der eigenen Kita

Der Mittelwert des zusammenfassenden standardisierten Summenindex aus den Hauptvariablen der oben beschriebenen 9 Bereiche plus 6 zusätzlicher Variablen (siehe Zusatzmaterial online) beträgt 0,82 (SD 0,16; Spanne: 0,3–1,0; möglicher Bereich 0–1: keine bis angemessene Organisation und Umsetzung des Arbeitsschutzes).

Zwei Drittel der 118 Befragten schätzten die Qualität des Arbeitsschutzes in ihrer Kita positiv ein (Schulnote 1 = sehr gut: 14,4 %, 2 = gut: 48,3 %). 27,1 % bezeichneten die Qualität als „befriedigend“ (Note 3), 7,6 % als ausreichend und insgesamt 2,5 % als „mangelhaft“ oder „ungenügend“.

Es besteht ein hoher und signifikant negative Zusammenhang zwischen dem Summenindex und der Selbstbewertung nach Noten (Spearman Rho = −0,64; *p* < 0,001): je besser der Summenindex, desto kleiner und damit besser der Notenwert.

### Wünsche und Verbesserungsvorschläge

Insgesamt 92 Befragte (76,6 %) äußerten sich in 156 Freitexten zur Frage nach besonderen Wünschen und Verbesserungsvorschlägen zum Arbeitsschutz (Tab. 6). An erster Stelle der Nennungen steht der Wunsch nach einer besseren bzw. ergonomischeren Ausstattung und generell eine bessere Organisation der Kita (27 %), dicht gefolgt vom Wunsch nach besseren und strukturierten Informationen zum Arbeitsschutz (25 %) und einer besseren Kommunikation zwischen dem Träger und der Kita zu Arbeitsschutzthemen (22 %).

**Table 6:** 

–	*N* (% von *n* = 92)
*Allgemeine Wünsche nach mehr Unterstützung*	
Bessere Kommunikation(sflüsse) zwischen Träger und Kita zum Arbeitsschutz	20 (21,7 %)
Mehr Unterstützung von Fachkraft für Arbeitssicherheit bzw. Betriebsarzt bzw. anderen externen Arbeitsschutzberater	18,5 (18,5 %)
Mehr Unterstützung vom Träger (unspezifische Aussage)	11 (12,0 %)
Mehr Unterstützung von der Unfallversicherung (unspezifische Aussage)	5 (5,4 %)
Mehr Engagement auf Kita-Ebene für den Arbeitsschutz^b^	2 (2,2 %)
*Spezielle Wünsche zum Arbeitsschutz*	
Bessere Informationen zum Arbeitsschutz (Materialien, z. B. in einem Ordner, Organisation)^c^	23 (25,0 %)
Mehr Fortbildungsmöglichkeiten zum Arbeitsschutz^d^	16 (17,4 %)
Mehr Unterstützung/Information zu bestimmten Arbeitsschutzthemen (außer Ergonomie und Lärmschutz)^e^	13 (14,1 %)
Mehr Angebote zur betrieblichen Gesundheitsförderung	4 (4,3 %)
*Ressourcenwünsche*	
Bessere bzw. ergonomischere Ausstattung/bessere Kita-Organisation^f^	25 (27,2 %)
Mehr Personal/besserer Betreuungsschlüssel	15 (16,3 %)
Mehr Zeit für Arbeitsschutzthemen^g^	4 (4,3 %)
Bessere Bezahlung/honorieren der Leistungen und des Aufwands der Sicherheitsbeauftragten	1 (1,1 %)

^a^Prozentuierungsbasis: Antwortende

^b^Freiwillige für die Aufgaben eines Sicherheitsbeauftragten; allgemein: bessere Umsetzung der Vorgaben in der Praxis

^c^Vorlagen mit Möglichkeit der individuellen Anpassung (für Unterweisungen, aber auch Checklisten mit zeitlichen Vorgaben, Verantwortlichen und Listen mit Dienstleistern, die für die Umsetzung klassischer Arbeitsschutzmaßnahmen beauftragt werden können). Übersicht der wichtigsten gesetzlichen Grundlagen/Regelungen/Qualifizierungsangebote (auch spezielle für neue Mitarbeiter), als Ordner oder im Intranet

^d^Mehrheitlich unspezifisch formuliert. Spezifische Vorschläge (KK, *n* = 3: Mutterschutz, Spezialthemen für Führungskräfte, Stressbewältigung; nonKK, *n* = 3: 2 Brandschutzhelferausbildung, Händehygiene, Erste Hilfe) + 1x mehr Erste-Hilfe-Gutscheine

^e^ Zu Gefährdungsbeurteilungen (*n* = 4), zu Unterweisungen (*n* = 2), zur Einführung eines Erinnerungssystems für bestimmte Arbeitsschutzaufgaben (*n* = 2), zur Intensivierung der arbeitsmedizinischen Angebotsvorsorge, zum SARS-CoV-2-Infektionsschutz (*n* = 2), zu Fragen der Ersten Hilfe (*n* = 2)

^f^Bauliche Verbesserungen, besserer Hitze- und Lärmschutz, mehr Erzieherstühle, bessere Organisation (kleinere Kindergruppen)

^g^Zum Teil Vorschläge zur Reorganisation von Ressourcen

## Diskussion

Zusammenfassend aus einer Bestandsaufnahme in 120 zufällig ausgewählten Kitas in überwiegend kirchlicher Trägerschaft sind die Anforderungen an eine angemessene *Organisation* des Arbeitsschutzes vielfach erfüllt, was sowohl die einzelnen Bereiche als auch der Summenindex-Kennwert zur Arbeitsschutzqualität verdeutlichen. Die Ergebnisse heben sich damit deutlich positiv von den Erkenntnissen anderer Studien ab, die kleine und mittlere Unternehmen (KMU) im Fokus haben.

In der Bestandsaufnahme von Sczesny et al. [12] wurde z. B. deutlich, dass ein erheblicher Teil befragter Inhaberinnen und Inhaber von KMU deutliche Wissensdefizite über gesetzliche Arbeitsschutzvorgaben hat. Defizite für die Organisation und Umsetzung von z. B. Gefährdungsbeurteilungen oder Unterweisungen von Beschäftigten können damit unmittelbar abgeleitet werden. Gleiches gilt für die Studien zum Arbeitsschutzwissens- und Umsetzungsstand von Amler et al. in KMU [1] und von der Gemeinsamen Deutschen Arbeitsschutzstrategie (GDA), in der ebenfalls u. a. der Umsetzungsgrad der Arbeitsschutzorganisation in KMU als defizitär bewertet wurde [8]. Auch ist der Anteil von Kitas mit einer betriebsärztlichen bzw. sicherheitstechnischen Betreuung höher im Vergleich mit Ergebnissen einer Interview-Pilotstudie in (anderen) kleineren/mittleren Betrieben [13]. Mögliche Gründe für die diskrepant positiven Befunde werden im Kapitel „Limitationen“ diskutiert.

Für eine gute *Umsetzung *von gesetzlich definierten formalen Arbeitsschutzvorgaben sind noch Entwicklungsmöglichkeiten erkennbar. Dies betrifft ...

- zum einen die *Aktivitäten von Arbeitsschutzakteuren*, also von Fachkräften für Arbeitssicherheit und Betriebsärzten (ihre Einbeziehung bei Gefährdungsbeurteilungen in kleinen und die Ermittlung ihrer Einsatzzeiten in größeren Kitas, ihre Beteiligung bei Gefährdungsbeurteilungen, aber im Gegenzug auch Transparenz durch schriftliche Berichte über die arbeitsmedizinische und sicherheitstechnische Betreuung);
- zum anderen die *Regelmäßigkeit *von Arbeitsschutzmaßnahmen wie Gefährdungsbeurteilungen, ASA-Treffen, Pflichtvorsorge, Unterweisungen, Qualifizierungsmaßnahmen und erforderliche Verbesserungen nach Arbeitsschutzkontrollen.

Die im Rahmen der Erhebung geäußerten Wünsche zu Verbesserungen des Arbeitsschutzes zeigen, dass ein Teil der Kita-Leitungen von einem besseren Zugang zu aktuellen Arbeitsschutzinformationen profitieren würden.

## Limitationen der Studie

Die Führungskräfte berichteten über das Ausmaß, inwieweit Arbeitsschutzregeln in ihrer Kita eingehalten werden, den Vertretern einer Institution, die für die Beratung und Überwachung des Arbeitsschutzes zuständig sind. Dies wirft zum einen die Frage auf, wie die Antworten ausgefallen wären, wenn statt Führungskräften die Beschäftigten selbst (z. B. Sicherheitsbeauftragte) oder bspw. Fachkräfte für Arbeitssicherheit befragt worden wären.

Zum anderen stellt sich die Frage, inwieweit die Leitungskräfte, die ihre Arbeit im Rahmen der vorliegenden Befragung ja selbst beurteilen sollten, in der persönlichen Befragungssituation gegenüber den Aufsichtspersonen der BGW in die Gefahr gerieten, Antworten im Sinne einer „sozialen Erwünschtheit“ [4] zu geben. Diese wird z. B. in Online-Befragungen als geringer angesehen [14]. Auf der anderen Seite bietet die durchgeführte Interviewsituation die Möglichkeit des gezielten Nachfragens, die Sichtung von Dokumenten und nicht zuletzt dank des gewählten Ansatzes die im Rahmen der Studie intendierte zeitgleiche Beratung bei Defiziten.

Zusammenfassend können mögliche Antwortverzerrungen in eine positive Richtung nicht ausgeschlossen werden. Zwar kann die hohe Korrelation mit der globalen Notenbewertung der Arbeitsschutzqualität in der eigenen Kita als Plausibilitätskontrolle herangezogen werden; da mit der Note aber wiederum die eigene Arbeit als Führungskraft selbst bewertet wird, sollte dieser Punkt jedoch ebenfalls kritisch betrachtet werden.

Die Daten wurden bis auf wenige Ausnahmen in Kitas kirchlicher Träger erhoben, also Einrichtungen, in denen vielfach Strukturen von *oben* vorgegeben sind. Es ist nicht bekannt, ob die Ergebnisse auf Einrichtungen öffentlicher und freier Träger ohne Weiteres übertragbar sind.

Die bislang fehlenden Informationen über die Sichtweisen der Kita-Beschäftigten selbst und die Frage der Übertragbarkeit der Ergebnisse auf andere Träger implizieren weiteren Forschungsbedarf.

## Ausblick

In den untersuchten Kitas wird ein Arbeitsschutzmanagementsystem deutlich seltener genutzt als in anderen Betrieben [1]. Hier sehen wir Unterstützungspotenzial, da zu vermuten ist, dass z. B. das Konzept des berufsgenossenschaftlichen *Arbeitsschutzes mit System* (das es auch angepasst an die Situation in Kitas gibt; [15]) oder andere Tools zur systematischen Organisation des Arbeitsschutzes [3] bzw. weitere Handlungsleitfäden hierbei eine positive Rolle spielen könnten. (Eine Übersicht bietet der Länderausschuss für Arbeitsschutz und Sicherheitstechnik LASI [9].)

Empfehlenswert wäre, in einschlägigen Kommunikationsmedien, bei der Beratung der Präventionsdienste in den Kitas, aber auch gegenüber den Arbeitsschutzverantwortlichen auf Trägerebene verstärkt auf diese Möglichkeit hinzuweisen und die Implementierung aktiv zu unterstützen. Zu fragen bleibt, an welchen Stellen die bei kirchlichen Trägern benannten Arbeitsschutzkoordinatoren ihre Kommunikation und Zuarbeit mit den einzelnen Kitas verbessern können. Dass hier Bedarf besteht, machen auf jeden Fall die Freitexte zu besonderen Wünschen deutlich.

Weiterer Forschungsbedarf besteht zu entsprechenden Erfahrungen aus einer Beschäftigtenperspektive und zum Arbeitsschutzstandard in nichtkirchlichen Einrichtungen.

## Finanzierung, Ethik und Danksagung

Die Studie wurde von der BGW finanziell unterstützt. Das Datenschutzkonzept wurde mit der Datenschutzbeauftragen der BGW abgestimmt. Ein Antrag bei der Ethikkommission einer Ärztekammer wurde nicht gestellt, da keine medizinischen Daten erhoben wurden. Die Autoren danken allen Beteiligten der BGW-Präventionsdienste für ihre Erhebungen und Birgit Eckert (FFAS) für die tatkräftige Organisation und das Datenmanagement.

## Fazit für die Praxis

- Der Organisationsgrad von Arbeitsschutzmaßnahmen in Kindertagesstätten (Kitas) mit mehrheitlich kirchlichen Trägern ist insgesamt recht hoch.
- Der Grad der tatsächlichen Umsetzung von Maßnahmen ist geringer, liegt aber nicht unter dem in anderen Branchen.
- Kita-Leitungen haben nicht zuletzt durch den anhaltenden Fachkräftemangel bekanntermaßen eine hohe Arbeitsbelastung und benötigen mehr Unterstützung für die Umsetzung von Arbeitsschutzmaßnahmen.
- Dies könnte durch eine intensivere Unterstützung der bei kirchlichen Trägern beauftragten Arbeitsschutzkoordinatoren erfolgen.
- Darüber hinaus sollten die bislang selten genutzten Arbeitsschutzmanagementsysteme mehr in Kitas implementiert werden.

## Supplementary Information




