# Supplementary material for: Organization and implementation of occupational health and safety in child day care centers
Source: Zentralbl Arbeitsmed Arbeitsschutz Ergon. 2022 Feb 4;72(3):99–106. [Article in German] doi: 10.1007/s40664-021-00454-6 (PMC8815014; doi:10.1007/s40664-021-00454-6)
Supplement: Supplementary file 1 [file 40664_2021_454_MOESM1_ESM.docx]

**Tabelle S1: Konstruktion eines standardisierten Summenindex**

|  | **Arbeitsschutzbereich ^a^** | **Codierung ^b^** |
| --- | --- | --- |
| 1. | Arbeitsschutzakteure schriftlich beauftragt bzw. bestellt bzw. angefordert bei Bedarf | 1.0 SiFa + Betriebsarzt; 0.5 SiFa (nur) oder Betriebsarzt; 0.0 kein Arbeitsschutzakteur beauftragt |
| 2. | Organisation von Gefährdungsbeurteilungen | 1.0 vollständig; 0.75 überwiegend; 0.25 überwiegend nicht; 0.0 gar nicht |
| 3. | Organisation der arbeitsmedizinischen Vorsorge | 1.0 vollständig; 0.75 überwiegend; 0.25 überwiegend nicht; 0.0 gar nicht |
| 4. | Schriftliche Benennung eines Sicherheitsbeauftragten ^c^ | 1.0 ja; 0.0 nein |
| 5. | Organisation Erste Hilfe |  |
|  | 1) Erste Hilfe organisiert | 1.0 vollständig; 0.75 überwiegend; 0.25 überwiegend nicht; 0.0 gar nicht |
|  | 2) Beschäftigte als Ersthelfer | 1.0 ja; 0.0nein |
|  | 3) Vorgehensweisen Erste Hilfe bekannt | 1.0 vollständig; 0.75 überwiegend; 0.25 überwiegend nicht; 0.0 gar nicht |
| 6. | Notfallmaßnahmen |  |
|  | 1) Notfallmaßnahmen organisiert | 1.0 vollständig; 0.75 überwiegend; 0.25 überwiegend nicht; 0.0 gar nicht |
|  | 2) Beschäftigte weitergebildet | 1.0 ja; 0.0nein |
|  | 3) Vorgehensweisen Notfälle bekannt | 1.0 vollständig; 0.75 überwiegend; 0.25 überwiegend nicht; 0.0 gar nicht |
| 7. | Unterweisungen organisiert | 1.0 vollständig; 0.75 überwiegend; 0.25 überwiegend nicht; 0.0 gar nicht |
| 8. | Qualifizierung Arbeitsschutz |  |
|  | 1) Qualifizierungsmaßnahmen durchgeführt | 1.0 regelmäßig; 0.5 unregelmäßig; 0.0 nie |
|  | 2) Kita-Leitung sind Arbeitsschutzpflichten bekannt ^d^ | 1ja; 0 nein |
| 9. | Arbeitsschutzkontrollen |  |
|  | 1) Kontrolle des Arbeitsschutz erfolgt regelmäßig | 1.0 vollständig; 0.75 überwiegend; 0.25 überwiegend nicht; 0.0 gar nicht |
|  | 2) Bei Bedarf erfolgen Verbesserungen | 1.0 immer; 0.75 meistens; 0.25manchmal; 0.0 nie |

Legende

Abkürzung: SiFa Sicherheitsfachkraft

^a^ Hauptvariablen aus neun Bereichen: Organisation von 1. Arbeitsschutzstrukturen, 2. Gefährdungsbeurteilungen, 3. arbeitsmedizinischer Vorsorge, 4. Sicherheitsbeauftragten, 5. Erste Hilfe-Maßnahmen, 6. Notfallmaßnahmen, 7. Unterweisung von Beschäftigen, 8. Arbeitsschutzqualifizierung von Beschäftigten, 9. Arbeitsschutzkontrollen plus

sechs zusätzliche Variablen: 5b. Ersthelfer ausgebildet, 5c. Vorgehensweisen der Ersten Hilfe sind bekannt, 6b. Brandschutzhelfer ausgebildet, 6c. Beschäftigten sind Vorgehensweisen bei Notfällen bekannt, 8b. der Kita-Leitung sind ihre Arbeitsschutzpflichten bekannt, 9b. Nach Arbeitsschutzkontrollen bei Bedarf Verbesserungen.

^b^ Möglicher Wertebereich: 0-1 keine bis angemessene Organisation bzw. Umsetzung des Arbeitsschutzes

^c^ Erforderlich bei >20 Beschäftigen; nahezu alle Kita erfüllen dies.

^d^ Aus diesem Bereich wurden zwei von drei Variablen verwendet (8.2 Qualifizierungsmaßnahmen durchgeführt und 8.3 Kita-Leitung sind Arbeitsschutzpflichten bekannt). Nicht verwendet: 8.1 Qualifizierungsbedarf ermittelt (Redundanz mit 8.2)
